# Urinary Immune Complexes Reflect Renal Pathology in Lupus Nephritis

**DOI:** 10.3390/diagnostics14242787

**Published:** 2024-12-12

**Authors:** Chenling Tang, Aygun Teymur, Tianfu Wu

**Affiliations:** Department of Biomedical Engineering, University of Houston, Houston, TX 77204, USA; ctang9@central.uh.edu (C.T.); arteymur@cougarnet.uh.edu (A.T.)

**Keywords:** immune complex, urinary biomarkers, lupus nephritis, machine learning, point-of-care testing

## Abstract

Background/Objectives: Lupus nephritis (LN) is a serious complication of systemic lupus erythematosus (SLE), involving immune complex deposition in the kidneys. While renal biopsy is the diagnostic gold standard, its invasiveness limits frequent use, driving the need for non-invasive urinary biomarkers to monitor disease progression and response to treatment. This study aimed to identify and validate urinary biomarkers for LN. Methods: Data from 10 LN-related omics databases, including urine, PBMCs, and kidney tissue, were analyzed. Differentially expressed proteins (DEPs) and genes (DEGs) were identified, and candidate biomarkers were validated via ELISA in an independent cohort of 87 urine samples. Results: We identified 78 biomarkers, with 14 overlapping across transcriptomic categories. Novel urinary biomarkers, including SERPING1, SLPI, and CD48, were validated. Urinary CD163, VCAM1, and ALCAM levels showed significant differences between LN and healthy controls, while urinary immune complexes (ICx) demonstrated superior diagnostic performance, with urinary ALCAM-ICx and CCL21-ICx achieving the highest AUC values. Conclusions: Our findings highlight the potential of urinary immune complexes and antigens as non-invasive biomarkers for LN. ALCAM, CD163, and SERPING1-ICx, in particular, were found as promising candidates for a urinary biomarker panel to aid in the diagnosis and monitoring of LN.

## 1. Introduction

Lupus nephritis (LN) is among the most serious manifestations of systemic lupus erythematosus (SLE), characterized by chronic inflammatory processes and the deposition of immune complexes within the kidneys [1]. This immune complex (ICx) accumulation provokes inflammation, leading to glomerular injury and progressive renal impairment. Antigens bind with antibodies to create these ICx, which can vary from small molecules to complex macromolecules or tissue components [2]. ICx can form continuously in response to infection, tissue damage, or foreign antigens, with the Fc regions of IgG activating the complement system [3]. In autoimmune diseases, these complexes interact with host tissue antigens, leading to immune-mediated tissue damage that exacerbates inflammation and contributes to disease pathology.

Nephritis is a frequent complication in patients with SLE, affecting between 14% and 55% of cases, with increased prevalence observed in Asian, African, and Hispanic populations [4]. Although renal biopsy remains the gold standard for LN diagnosis and evaluation, it is invasive, carries a risk of complications, and is not suitable for frequent disease monitoring [5]. In addition, renal biopsy offers only a limited view of disease activity at a single point in time and may fail to detect focal lesions.

Consequently, there has been an increasing focus on discovering non-invasive urinary biomarkers capable of reflecting the underlying renal pathology, monitoring disease progression, and predicting response to treatment. Urinary biomarkers have the potential to capture early changes in renal pathology, often preceding the onset of clinical symptoms or significant proteinuria [6]. This early detection capability not only enables timely intervention and more proactive management of LN but also provides real-time insights into kidney function, minimizing the need for repeated invasive procedures.

The ideal urinary biomarker should accurately reflect both the activity of the underlying renal pathology and the overall severity of the disease. Such a biomarker would provide crucial insights into disease dynamics, offering a reliable means to assess ongoing renal damage while also serving as an indicator of disease burden. These markers include neutrophil gelatinase-associated lipocalin (NGAL), monocyte chemoattractant protein-1 (MCP-1), kidney injury molecule-1 (KIM-1), and tumor necrosis factor-like weak inducer of apoptosis (TWEAK), all of which have been found to correlate with active LN [7,8]. However, more recent studies have highlighted novel candidates such as urinary immune complexes, vascular cell adhesion molecule-1 (VCAM-1), and angiostatin, which show promise in improving the diagnosis and management of LN [9]. The formation and deposition of immune complexes (ICx) within renal tissues is a defining feature of LN. This deposition triggers a cascade of inflammatory responses, complement activation, and ultimately leads to kidney damage. Due to their pivotal role in LN pathogenesis, urinary ICx have garnered attention as potential biomarkers for tracking disease activity [10]. The use of urinary immune complexes as biomarkers is supported by their direct involvement in the underlying mechanisms of renal injury in LN. The initial glomerular damage caused by immune complexes differs depending on the location of their deposition within the kidney [11]. Notably, urinary immune complexes may provide greater specificity for LN compared to traditional biomarkers, as their elevation is more directly associated with immune complex-mediated kidney injury. This distinction sets them apart from markers commonly used for chronic kidney disease (CKD), where immune complexes play a less prominent role.

In recent years, the advent of omics technologies has revolutionized the field of biomarker discovery. Omics encompasses the large-scale study of biological molecules, such as proteins, genes, and metabolites, and allows for the systematic analysis of complex biological processes across multiple datasets [12]. These technologies, including proteomics, genomics, and transcriptomics, provide comprehensive insights into disease mechanisms at a molecular level, identifying patterns and changes that might not be apparent from clinical observations alone. By analyzing large omics datasets, novel biomarkers can further be linked to disease pathogenesis, offering a pathway to more personalized diagnostics and treatment strategies. In addition, the integration of omics data from multiple sources offers a powerful solution to the challenges of small cohort sizes and variability in disease progression. The ability to access and analyze these datasets enables the investigations into molecular targets and biomarkers of LN at a scale that would be unattainable with traditional sample collection alone. This approach not only increases statistical power but also enables the identification of biomarkers that are more robust across different populations and clinical settings.

In our study, we identified 10 LN-related public omics databases to investigate urinary biomarkers with high potential for diagnosing and monitoring LN. By integrating data from multiple cohorts, we were able to evaluate a wide variety of molecular targets, ultimately validating protein targets and creating a biomarker panel that could potentially be used to aid in LN diagnosis. This comprehensive analysis allowed us to overcome the limitations of small sample sizes and variability in disease presentation, providing a more robust foundation for the identification of novel urinary biomarkers in LN.

## 2. Materials and Methods

### 2.1. Patients and Clinical Samples

Serum samples from LN, chronic kidney disease (CKD), and healthy controls (HC) were collected at the University of Texas, Southwestern Medical Center at Dallas. All human subject-related procedures were performed following the University of Houston-approved IRB protocols, and the consents of all subjects were obtained before sample collection. The detailed demographics and clinical information are summarized in Table 1. All human urine samples were aliquoted upon receipt and stored at −80 °C. The flare of lupus nephritis (LN flare) was defined with the following three criteria: (1) the urine protein/creatinine ratio (uPCR) > 0.5 (mg/mg). (2) serum creatinine increase > 15–20% of the baseline (3) > 5 RBC/HPF in urine, otherwise was defined as remission lupus nephritis (LN-Remission) [13]. The matched samples from CKD and healthy control groups were tested along with those from LN. CKD patients were utilized as a disease control to account for general renal pathology.

### 2.2. LN-Related DEGs and DEPs in 10 Omics Databases

Ten publicly available LN-related omics databases (Table 2) were downloaded to identify potential urine biomarkers of LN. In the ten databases, data from three sample types (urine, PBMCs, and kidney tissue) and five different technologies: aptamer-based protein array, antibody-based protein array, gene expression microarray ( expression array), total RNA sequencing (RNA-seq), and single-cell RNA sequencing (scRNA-seq) were included to eliminate bias introduced from specific technologies, sample types or sample cohorts. Among the data from two urinary proteome array studies, the DEPs with area under the curve (AUC) ≤ 0.95 were filtered out. Due to the complexity of the renal samples and challenges in cell isolation [14], five kidney databases (bulk RNA-seq or RNA-array) were combined, and only DEGs found in two or more databases were retained for further analysis. The DEGs/DEPs were retrieved with cutoffs listed in Table 2 and corresponding R tools, including Seurat, DESeq2, GEOquery, and Limma [15,16,17]. Candidate biomarkers were selected based on if they were present in both the genome database and the proteome database while satisfying the cutoff criteria shown in Table 2. The PPI network was constructed by mapping the candidate biomarkers (preferably present at least in 2 databases) to the Search Tool for the Retrieval of Interacting Genes (STRING) [16] with the full STRING network and interaction score larger than 0.40.

### 2.3. Independent Validation Studies Using ELISA

The omics co-occurrence and literature-searched biomarkers were validated using commercially available ELISA kits in an independent validation study using a cohort of 87 (53 LN-Flare, 8 LN-Remission, 13 CKD, and 13 HC) urine samples. The absolute level of urine proteins was determined using standard curves on each ELISA plate with an optimized urine dilution ratio. SERPING1, SLPI, VCAM1, ALCAM, NGAL, BAFF, CCL21, TFPI, CD163, and SPP1 were measured with DuoSet ELISA kits purchased from R&D Systems (Minneapolis, MN, USA), and VSIG4, CFL1, MSN, and CD48 were measured with ELISA kits purchased from RayBiotech (Peachtree Corners, GA, USA).

Corresponding immune complexes (ICx) to these biomarkers were measured using in-house-developed ELISA kits. The ICx assays utilized capture antibodies obtained from the same source as those used in commercial ELISAs. These antibodies were employed to capture antigen–antibody complexes, which were subsequently detected using labeled anti-human IgG. Briefly, monoclonal antibodies were purchased from R&D Systems (Minneapolis, MN, USA) and RayBiotech (Peachtree Corners, GA, USA) and used to coat the plate to measure the urine ICx. Then, anti-human IgG antibody (Jackson ImmunoResearch, West Grove, PA, USA) was diluted 1:20,000 and incubated for 2 h at room temperature, followed by color development with streptavidin-HRP and TMB substrate. ELISA signals were read using an Epoch plate reader (BioTek Instruments, Winooski, VT, USA) at 450 nm, and the 4-parameter logistic (4PL) standard curves were generated based on serially diluted standards to determine the protein concentrations of the antigen form in the urine. Urinary creatinine levels of all samples were measured with the Creatinine Parameter Assay Kit (R&D Systems, Minneapolis, MN, USA) for normalizing the biomarker levels in urine.

### 2.4. Statistical Analysis

All data were analyzed, plotted, or visualized with R 4.1.0 language or ggplot2 package [28]. Group-wise significant differences were determined by the Wilcoxon test. AUC analysis was performed using a pROC package [29]. The correlation between biomarker levels and clinical or pathological parameters was determined using Spearman’s correlation coefficient and the COR package [30], and their interchangeable relationship was measured using a linear regression model. The potential disease diagnosis (HC vs. LN classification) with six machine-learning models was trained with SciKit-learn and plotted with matplotlib from Python 3.7.6 [31].

## 3. Results

### 3.1. Discovery of Novel Urine Biomarkers in Lupus Through Omics Analysis

**Figure 1:**
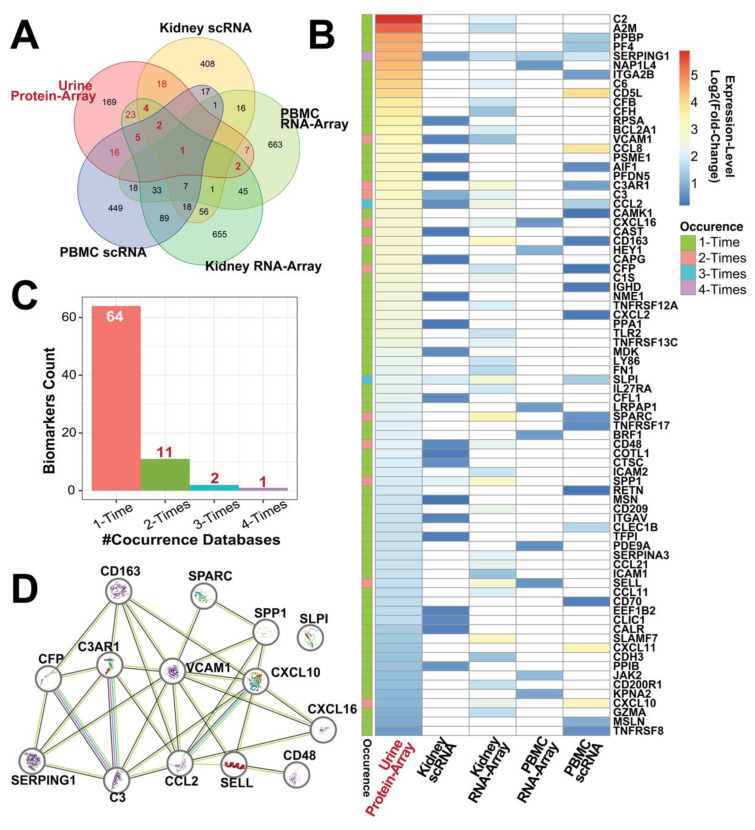
Omics Identification of LN concurrence biomarkers. (**A**) Venn diagram showing overlap of differentially expressed proteins (DEPs) and genes (DEGs) from urine protein array, kidney scRNA, kidney RNA-array, PBMC scRNA, and PBMC RNA-array datasets. (**B**) Heatmap displaying the expression levels (log2 fold change) of selected biomarkers, colored based on their frequency of occurrence across the databases. (**C**) Bar graph depicting the number of biomarkers with co-occurrence in 1 to 4 datasets. (**D**) Protein–protein interaction network of top DEPs/DEGs identified, visualized using STRING analysis.

The ten databases can be grouped into five categories based on the study subjects and screening technologies as shown in Figure 1A: urine protein array, kidney scRNA, kidney bulk RNA-Seq, PBMC scRNA, and PBMC RNA microarray.

In this study, we focused on the urine biomarkers with genomic hits; thus, the 78 biomarkers (colored in red in Figure 1A) that overlapped in proteomic and genomic major categories were selected, and their expression levels were displayed in a heatmap (Figure 1B). As Figure 1C indicates, 14 biomarkers were found to appear in more than two transcriptomic categories. Notably, SERPING1 is the only biomarker with co-occurrence in all four transcriptomic categories while also ranking the 4th highest at the fold-change ratio in urine protein expression. Except for SELL and SPARC, the other 12 genes belong to the defense response biological process (FDR < 0.0001), and CFP, SERPING1, C3, SLPI, CCL2, and CXCL10 are involved in the humoral immune response process (FDR < 0.0001) which may suggest the impaired humoral memory responses to early-present antigens in kidney disease [32]. VCAM1 is in the center of this PPI network, combined with CCL2 and CXCL10 together to contribute to the TNF signaling pathway (FDR = 0.012).

Among the 14 identified proteins, SERPING1, SLPI, and CD48 are novel urine biomarkers in lupus nephritis. The remaining 11 have already been found elevated in LN urine compared to healthy patients and been discussed extensively in previous studies [7,18,19,33,34,35,36,37,38,39,40]. To construct a biomarker panel, the OPN, CD163, and VCAM1 were chosen to test side by side with novel biomarkers. Although the CFL1 and MSN each showed one co-occurrence in our analysis, the peer studies demonstrate they are associated with the pathological mechanisms in autoimmune diseases [41,42,43]. Additionally, ALCAM, NGAL, BAFF, CCL21, TFPI, and VSIG4 were reported to contribute to LN diagnosis and were added for further validation [44,45,46,47,48].

### 3.2. Validation of 17 Omics-Derived Screening Targets in Lupus Nephritis

An independent cohort of 87 subjects (53 LN-Flare, 8 LN-Remission, 13 CKD, and 13 HC) was used for ELISA validation. With a urine dilution of 1:5, the CFL1, MSN, CD48, BAFF, CCL21, TFPI, SLPI, and VSIG4 were below the lowest limit of detection for our samples; thus, their urine antigen data were excluded from further analysis.

**Figure 2:**
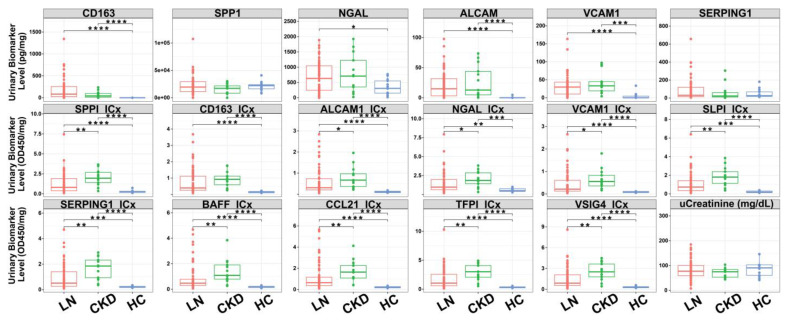
ELISA screening of potential urine biomarker candidates of LN. Box plots showing the results of ELISA validation for selected urinary antigens (top row) and their corresponding immune complexes (ICx, middle and bottom rows) across lupus nephritis (LN), chronic kidney disease (CKD), and healthy control (HC) groups. Biomarkers analyzed include CD163, SPP1, NGAL, ALCAM, VCAM1, SERPING1, BAFF, CCL21, TFPI, VSIG4, SLPI, and uCreatinine. All urinary biomarker levels (shown in Y-axis) were normalized by urinary creatinine. Statistical significance between groups is indicated by asterisks (* *p* < 0.01; ** *p* < 0.001; *** *p* < 0.0001; **** *p* < 0.00001).

In the urine antigen measurement (Figure 2, row 1), SPPI and SERPING1 show no significant difference in group-wise comparison, whereas CD163, VCAM1, and ALCAM show significant differences between diseases (both LN and CKD) with healthy groups. However, no significance was found in the LN and CKD comparison. Overall, the urine ICx (Figure 2, row 2 and row 3) showed stronger and similar group-wise differences compared to urine antigen, which may be related to the impaired kidney function leaking the deposited various types of immune complex into urine [49]. The urine ICx analysis demonstrated superior performance compared to the clinical urine creatinine parameter.

### 3.3. Diagnostic Potential of Urinary Antigens and ICx

**Figure 3:**
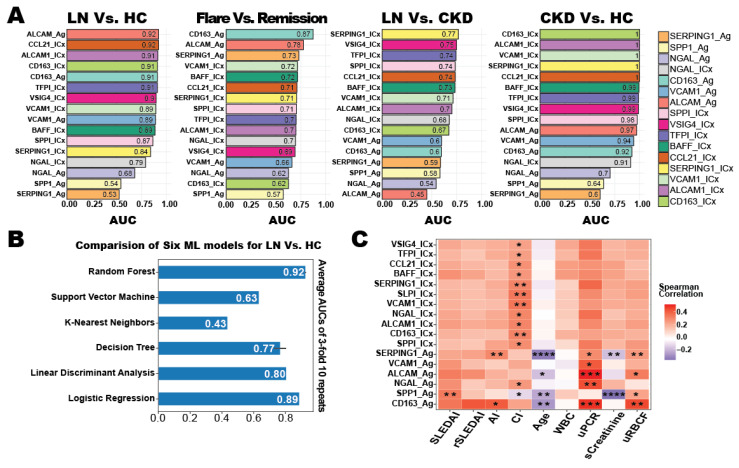
Diagnostic potential of urinary antigens and ICx. (**A**) Bar graphs displaying the area under the curve (AUC) values for individual biomarkers and immune complexes (ICx) in distinguishing LN from healthy controls (HC), LN flare from remission, LN from CKD, and CKD from HC. (**B**) Comparison of six machine-learning models for classifying LN versus HC, showing the average AUC values from 3-fold, 10-repeats. Models include random forest, support vector machine, K-nearest neighbors, decision tree, linear discriminant analysis, and logistic regression. (**C**) Heatmap illustrating the Spearman correlation between urinary biomarkers and clinical/pathological indices, including SLEDAI, AI (activity index), CI (chronicity index), age, WBC, uPCR, serum creatinine, and uRBC. Statistical significance is denoted by asterisks (* *p* < 0.01; ** *p* < 0.001; *** *p* < 0.0001; **** *p* < 0.00001).

To evaluate the diagnostic performance of the above urine antigen and ICx in distinguishing LN versus HC, LN-Flare versus LN-Remission, LN versus CKD, and CKD versus HC, we individually applied area under the curve (AUC) analysis for each biomarker and plotted their AUC values in each paired comparison (Figure 3A). ALCAM (AUC = 0.92) and CCL21-ICx (AUC = 0.92) had better diagnostic capabilities when distinguishing between LN and HC, while CD163 (AUC = 0.87) and ALCAM (AUC = 0.78) had the best AUC values for distinguishing between LN flare or remission statuses. For distinguishing kidney-specific diseases, SERPING1-ICx (AUC = 0.77) and VSIG4-ICx (AUC = 0.75) had better performance out of all of the other markers to differentiate LN from CKD. Remarkably, bound-form ICx biomarkers were better at distinguishing CKD from LN and HC than their free-form overall.

Using machine learning, integrating biomarkers with unique characteristics into a biomarker panel has been demonstrated to improve lupus diagnosis [50,51]. Six machine-learning models with different learning principles (linear model: linear discriminant analysis, and logistic regression; non-parametric models: K-nearest neighbors, decision trees; ensemble models: random forests; kernel-based models: support vector machines) were trained to solve the LN versus HC classification problem [52]. However, the machine models were not able to improve the diagnostic performance compared with the ROC analysis (Figure 3B). In the clinical and pathological index correlation analysis, SERPING1 was found positively correlated with AI, urine PCR, and urine RBC/HPF, and negatively correlated with age at sampling and serum creatinine (Figure 3C). The urine ICx results display a significant positive correlation with the chronicity index (CI), which consists of our previous serum ICx study [53]. By integrating the performance of ROC in different paired comparisons and clinical correlation analyses, ALCAM (top AUC in LN vs. HC and strongly correlated with uPCR), CD163 (top AUC in LN-Flare vs. LN-Remission and strongly correlated with uPCR and uRBC), SERPING1-ICx (top AUC in LN vs. CKD and strongly correlated with AI and uRBC), and SPP1 (strongly correlated with SLEDAI, CI, and sCreatinine) were identified as candidates for an LN urine biomarker panel.

## 4. Discussion

In this study, we aimed to identify and validate urinary biomarkers for LN by utilizing multi-omics data from 10 public databases, enabling us to analyze proteomic and genomic overlaps across different cohorts. This approach enabled us to overcome a primary obstacle in biomarker discovery for LN, namely the challenge of acquiring large, well-characterized patient cohorts. By integrating omics data from various study types, we identified 78 potential biomarkers, 14 of which were identified as having significant expression across multiple transcriptomic categories. Notably, a potentially novel LN marker, SERPING1, is the only biomarker with co-occurrence in all four transcriptomic categories while also ranking the 4th highest at the fold-change ratio in urine protein expression.

The validation of these omics-derived candidates in an independent cohort of 87 subjects, including LN, CKD, and HC patients, revealed several key findings. Although several of the identified biomarkers (e.g., CFL1, MSN, BAFF, CCL21, TFPI, SLPI, and VSIG4) were below the detection limit in our ELISA validation, biomarkers such as CD163, VCAM1, and ALCAM showed significant differences between disease and healthy groups. However, it is noteworthy that these markers did not differentiate LN from CKD. This suggests that while these proteins are elevated in disease states, their expression may not be specific to LN, indicating that further investigation is necessary to improve disease specificity.

Interestingly, ALCAM (activated leukocyte cell adhesion molecule) has been studied in other autoimmune diseases and has been linked to tissue damage and inflammation. Tissue expression of ALCAM was seen to be upregulated in response to proinflammatory cytokines such as TNF-α and IFN-γ [54]. In the context of SLE, ALCAM has been found to be upregulated in patients with active disease, particularly in those with nephritis [48]. VCAM1 (vascular cell adhesion molecule 1) has similarly been identified as a mediator of inflammation and has been shown in other studies to correlate with disease activity in LN, promoting immune cell infiltration into renal tissue [33,55]. Our findings support these previous observations, as both ALCAM and VCAM1 demonstrated significant differences between LN and HC patients.

The ICx tested in our study exhibited a stronger diagnostic performance compared to urinary antigen levels, suggesting that they may be more reflective of the ongoing immune-mediated damage in LN. ICx levels have previously been shown to correlate with disease flares in SLE, especially in patients with nephritis [56,57]. Our results corroborate this finding, as ICx biomarkers showed stronger group-wise differences and better performance in distinguishing LN from both CKD and HC.

The diagnostic performance of individual biomarkers, evaluated through ROC analysis, further underscores the potential of ICx biomarkers in distinguishing disease states. While ALCAM and CCL21-ICx had the highest AUC values for distinguishing LN from HC (AUC = 0.92), a novel LN biomarker, SERPING1-ICx, also demonstrated a high AUC value of 0.84 in separating LN from HC. SERPING1 has been found to prevent the activation of the classical pathway on immune complexes, particularly when the antibody has a low affinity for the antigen [58]. It has been observed to prevent complement activation by inhibiting C1 complex binding with IgG [59], suggesting that it could contribute to LN by dislodging the activated C1 complex from immune complexes in the kidneys, attempting to reduce complement activation, and limiting immune-mediated renal damage. SERPING1’s presence in LN has been less frequently discussed, although a genetic mutation in the SERPING1 gene has been investigated in patients with active SLE [60], linking it to the regulation of complement pathways that are frequently dysregulated in LN. In our study, SERPING1 emerged as a promising candidate, particularly in its immune complex form. Similarly, CCL21, previously identified in the literature as a serum biomarker with high sensitivity for SLE patients with pulmonary involvement [61], shows promise in our study, particularly in its immune complex form (CCL21-ICx). CCL21, a chemokine involved in the recruitment of T cells and dendritic cells to inflamed tissues, plays a crucial role in immune cell trafficking and has been implicated in the chronic inflammation characteristic of autoimmune diseases [62].

CD163 outperformed other markers in distinguishing LN-Flare from LN-Remission (AUC = 0.87), highlighting its potential as a marker for disease activity. CD163, a scavenger receptor expressed on macrophages, has been identified as a marker of monocyte and macrophage activation, and its soluble form has been linked to inflammation in various autoimmune conditions [63,64]. A previous study has demonstrated elevated levels of soluble CD163 in LN patients, with significant correlations with renal SLEDAI, and it could potentially behave as a histological biomarker that correlates with CD163 cells in the glomeruli [65]. Our findings confirm that CD163, particularly in its immune complex-bound form, could serve as a robust marker for distinguishing disease activity in LN.

These proteins can exist in two forms, free-form antigens and bound-form immune complexes (ICx), with their relative levels potentially varying under different physiological or disease conditions. These variations may reflect underlying pathological processes, as the balance between free-form antigen and ICx is influenced by factors such as immune activation, antigen availability, and the presence of inflammatory responses. For instance, LN conditions may lead to elevated ICx levels being generated and excreted into the urine, while free-form antigen levels remain unchanged. The detectability of these forms can be influenced by variations in assay sensitivity and limits of detection. Antigen detection typically employs a sandwich ELISA format using an antigen-specific capture antibody, whereas ICx detection utilizes anti-human IgG to target the antibody component of the ICx, potentially providing greater sensitivity for identifying the ICx.

The integration of machine-learning methods in this study aimed to explore advanced analytical approaches for improving diagnostic precision in LN. Machine learning offers the potential to process complex, multidimensional data and identify subtle patterns that may not be evident through traditional statistical methods. By incorporating multiple models with distinct learning principles—ranging from linear models to non-parametric and ensemble methods—we sought to assess whether such approaches could enhance biomarker classification and provide deeper insights into disease stratification. Although the machine-learning models did not significantly outperform the ROC analysis in this case, their application underscores the value of exploring computational techniques that can handle large datasets with greater complexity. This may indicate that while these biomarkers are informative, further refinement and possibly the addition of more diverse biomarkers are required to capture the full complexity of LN pathogenesis. Nonetheless, combining ROC performance with clinical correlation allowed us to construct a more refined biomarker panel. ALCAM, CD163, SERPING1-ICx, and SPP1 were selected based on their diagnostic potential and strong correlations with clinical indices, such as uPCR, AI, and uRBC. SPP1 (secreted phosphoprotein 1), also known as osteopontin, has previously been implicated in SLE progression, suggesting genetic variants in the SPP1 gene that are correlated with SLE patients [66,67].

These machine-learning methods remain promising tools for future research, particularly in refining biomarker panels, improving disease classification, and predicting patient outcomes in autoimmune diseases like LN. Moving forward, continued development of these models, combined with larger datasets, may unlock their full potential in enhancing diagnostic accuracy and personalization in LN management.

The current LN urine biomarker panel is far from complete. The performance of this panel can be improved in disease diagnosis, stratification, and monitoring by incorporating the novel urine biomarkers. While previously reported markers, such as Kim-1, NGAL, and IL-18, have demonstrated good detectability and performance using ELISA, their absence among the top candidate genes identified in the transcriptome analysis may be attributed to discrepancies between gene and protein expression levels. As gene expression does not always correlate with protein expression due to post-transcriptional processing, post-translational modifications, and other regulatory mechanisms, it is plausible that these biomarkers, although effective in protein-based assays, were not prominent in transcriptomic analyses. Transcriptomic data provide valuable insights for identifying potential biomarkers of LN. However, top candidates identified through transcriptomics often fail to translate into reliable or validated protein biomarkers for LN. This highlights the need for more comprehensive and high-quality proteomics databases to facilitate the discovery, identification, and validation of novel protein biomarkers. Advanced computational approaches, including machine learning and deep learning, hold significant promise for accelerating biomarker discovery in LN. Despite their potential, the relatively small sample sizes commonly available in current LN studies present a challenge. Robust machine-learning models typically require large datasets to achieve reliable and generalizable results. Addressing this limitation is critical to unlocking the full potential of these technologies in LN biomarker research.

Although our study was limited by the sample size, especially with remission LN samples, SERPING1 may provide stronger flare versus remission differentiation capability as it significantly correlated with renal pathology activity index (AI), uPCR, and uRBC. Obtaining a substantial number of patient samples, particularly those representing different classes of LN, would enable a more robust multi-omics approach to identify reliable biomarkers with enhanced statistical power. Nonetheless, assembling such a cohort can be challenging, as it would likely require a multi-center effort over several years to acquire an adequate number of samples, especially from well-characterized LN populations. To address this challenge, we employed an alternative strategy by leveraging the integration of real-world clinical data with multi-omics datasets. This approach, previously shown to be effective in biomarker discovery, is both practical and powerful in situations where large patient cohorts are not readily available. While this approach utilizes diverse datasets, it may introduce variability due to differences in sample collection protocols, processing methods, and patient populations across the included studies. Additionally, the focus on biomarkers present in multiple databases with predefined cutoff criteria may have excluded potentially significant markers specific to smaller or less represented cohorts. This study was also limited to a cross-sectional analysis, which did not allow for the evaluation of disease progression or treatment response over time.

Future research will focus on the characterization and further validation of the ICx biomarkers, specifically in assessing the clinical implications of ICx levels in disease progression with a larger cohort. This includes investigating the mechanisms driving their formation and clearance from the body by evaluating their behavior across different LN disease stages and patient subgroups. Given the superior performance of the ICx form compared to the antigen form, further studies should explore the potential interactions of ICx with other immune components to better understand their role in disease pathogenesis. Additionally, the integration of advanced machine-learning algorithms with enhanced feature selection techniques could further be implemented to identify the most significant biomarkers and improve predictive modeling.

Although we limited our omics database search within the human species, the murine species may be a resource for novel biomarker discovery, especially genetically modified mouse models like NZB/W F1, MRL/lpr, and BXSB/Yaa [68]. To meet the rapid and efficient requirements of point-of-care testing and precision diagnosis, this urine biomarker panel can be incorporated into a one-step lateral flow microarray immunoassay. This immunoassay would have the shared array merits of the accuracy of quantitative multiplex and the easy use and rapid characterization of lateral flow assays [69].

## 5. Conclusions

In conclusion, our findings highlight the potential of immune complexes, particularly urinary CD163-ICx and SERPING1-ICx, as robust biomarkers for LN. We identified ALCAM, CD163, SERPING1-ICx and SPP1 as potential candidates in a urine biomarker panel. The identification of these markers, combined with previous studies confirming their roles in LN and other autoimmune diseases, underscores the importance of further validating these biomarkers in larger, more diverse cohorts. Future studies should focus on refining this biomarker panel and investigating the role of immune complexes in the pathophysiology of LN to improve disease monitoring and treatment outcomes.

## Figures and Tables

**Table 1:** Demographics and clinical characteristics of patients and health controls.

Lupus Nephritis Patients	LN-Flare	LN-Remission	Mann–Whitney *p*-Value
Total no. of subjects	53	8	/
Female (%)	90.57%	100.00%	/
Age, mean ± SE., years	26.60 ± 7.40	34.24 ± 13.54	0.32
Ethnicity, Asian/Black/Hispanic/White, no.	9/22/14/18	1/1/0/11	/
SLEDAI, median (interquartile)	10 (8–14)	5 (1.5–9)	1.20 × 10^−2^
Renal SLEDAI, median (interquartile)	8 (4–8)	0 (0–4)	<0.0001
AI, median (interquartile)	6 (2.0–8.0)	2.5 (1.0–4.25)	0.21
CI, median (interquartile)	3 (1.0–5.75)	1.5 (0–2.25)	0.09
Serum creatinine, mg/dl, median (interquartile)	3.30 (1.67–6.04)	0.20 (0.10–0.22)	0.29
Urine protein:creatinine ratio, mg/mg, median (interquartile)	2.91 (1.43–5.31)	0.20 (0.18–0.30)	<0.0001
Chronic Kidney Disease Patients			
Total no. of subjects			13
Female (%)			53.84%
Age, mean ± SE., years			40.08 ± 12.77
Ethnicity, Asian/Black/Hispanic/White, no.			1/7/0/5
Serum creatinine, mg/dl, median (interquartile)			1.63 (0.94–2.50)
Urine protein:creatinine ratio, mg/mg, median (interquartile)			3.02 (1.40–5.30)
CKD Stage, 2/3/4/5, no.			5/5/2/1
Healthy Controls			
Total no. of subjects			13
Female (%)			53.85%
Age, mean ± SE., years			26.87 ± 6.67
Ethnicity, Asian/Black/Hispanic/White, no.			0/7/0/6

**Table 2:** SLE/LN Omics databases used for discovery of candidate biomarkers of LN.

ID	Database Type	Citation	Accession Number	# Lupus	# HC	DEG/DEP Cut-off	# DEGs/DEPs	# Total Genes
1	Urine protein-array (Somalogic)	Stanley_2020 [18]	/	7	8	AUC > 0.95	247	1129
2	Urine protein-array (RayBiotech)	Vanarsa_2020 [19]	/	15	9	AUC > 0.95	247	1000
3	Kidney scRNA	Hansen_2022 [20]	10.48698/92nk-e805	12	20	FC > 1.5 and *p*-value < 0.05	549	30,395
4	Kidney RNA-array	Grayson_2018 [21]	GSE104948	32	21	FC > 1.2 and *p*-value < 0.05	128	12,040
5	Kidney RNA-seq	Park_2022 [22]	GSE175759	3	22	FC > 1.5 and *p*-value < 0.05	1516	42,001
6	Kidney RNA-array	Parikh_2022 [23]	GSE200306	58	10	FC > 1.2 and *p*-value < 0.05	116	527
7	Kidney RNA-array	Berthier_2012 [24]	GSE32591	32	15	FC > 1.2 and *p*-value < 0.05	301	12,029
8	Kidney RNA-seq	Yao_2021 [25]	GSE157293	3	3	FC > 1.2 and *p*-value < 0.05	697	25,725
9	PBMC scRNA	Belaid_2020 [26]	Phs002048.v1.p1	8	3	FC > 1.5 and *p*-value < 0.05	656	8406
10	PBMC RNA-array	Bienkowska_2014 [27]	GSE45291	292	20	FC > 1.5 and *p*-value < 0.05	794	54,715

## Data Availability

The original contributions presented in this study are included in the article. Further inquiries can be directed to the corresponding author.
